# Identification and Validation of an Immune Evasion Molecular Subgroup of Patients With Colon Cancer for Implications of Immunotherapy

**DOI:** 10.3389/fgene.2022.811660

**Published:** 2022-08-05

**Authors:** Hongbin Zhang, Zaifa Hong, Peipei Li, Han Jiang, Pengfei Wu, Jinzhong Chen

**Affiliations:** ^1^ Endoscopy Center, The First Affiliated Hospital of Xiamen University, School of Medicine, Xiamen University, Xiamen, China; ^2^ Department of Hepato-Biliary-Pancreatic and Vascular Surgery, The First Affiliated Hospital of Xiamen University, School of Medicine, Xiamen University, Xiamen, China; ^3^ Department of Hepato-Biliary-Pancreatic Surgery, Xiamen Hospital, Beijing University of Chinese Medicine, Xiamen, China; ^4^ Department of General Surgery, The First Affiliated Hospital of Xiamen University, School of Medicine, Xiamen University, Xiamen, China; ^5^ Department of General Surgery, The Second Affiliated Hospital of Soochow University, Suzhou, China

**Keywords:** colon cancer, immune evasion, prognosis, immunotherapy, signature

## Abstract

Immune evasion (IEV) plays a critical role in the development and progression of colon cancer. However, studies to predict the prognosis of colon cancer via IEV-related genes are limited. Therefore, based on the 182 IEV-related genes, we used the univariate and Lasso Cox regression model to construct the IEV-related genes signature (IEVSig) of 16 prognostic IEV-related genes using the Gene Expression Omnibus and The Cancer Genome Atlas online databases. We found that IEVSig was an independent prognostic factor, and patients with high IEVSig had higher TNM stage and shorter recurrence-free survival than their counterparts. Kyoto Encyclopedia of Genes and Genomes and gene set enrichment analyses revealed that patients with high and low IEVSig had significantly different enrichment pathways. Immune cell infiltration analysis showed that nine immune cells obviously increased in the high-IEVSig group, whereas five immune cells increased in the low-IEVSig group. Immunotherapy cohort analysis revealed that patients with high IEVSig had a higher proportion of progressive disease or stable disease after receiving immunotherapy than patients with low IEVSig. Furthermore, patients with low IEVSig had higher tumor mutation load and neoantigen burden, which indicated an improved response to immunotherapy, than patients with high IEVSig. Thus, an IEV-related prognostic signature was established to predict the prognosis of patients with colon cancer and derive a prediction marker to offer insights into therapeutic strategies.

## Introduction

Colon cancer is the third leading cancer in terms of incidence but second in terms of mortality worldwide (Bray et al., 2018). Given the high mortality of colon cancer, searching for and establishing effective biomarkers is a significant task. At present, the evaluation of prognosis includes the tumor stage, tumor anatomical location, and microsatellite status of tumors (Dekker et al., 2019). However, the complexity associations among biomarkers, patient prognosis, and treatment benefits make the management of patients with colon cancer challenging (Sveen et al., 2020). Therefore, a novel and effective prognostic assessment model must be urgently established.

The tumor microenvironment (TME) plays a crucial role in the development and progression of tumors. The TME is mainly composed of tumor cells, immune cells, and stromal cells. In tumor immune surveillance, the major cytotoxic lymphocytes, CD8^+^ T cells and natural killer (NK) cells, can direct perforin-dependent tumor cell killing and release several inflammatory cytokines, such as IFN-γ and TNF, to promote antitumor immunity through antigen presentation (Voskoboinik et al., 2015; Kearney et al., 2017). Major histocompatibility complex (MHC)-I-mediated antigen presentation facilitates the detection of tumor cells through cytotoxic CD8^+^ T cells. Therefore, disruption of antigen presentation is a key mechanism of tumor IEV (Kearney et al., 2018; Freeman et al., 2019). For example, loss-of-function mutations in B2M, JAK1, and JAK2, resulting in loss of MHC-I expression (B2M) or response to IFN-γ (JAK1 and JAK2), have been identified in patients who fail to respond to immunotherapy (Restifo et al., 1996; Zaretsky et al., 2016; Shin et al., 2017). In addition, the activation of other specific gene expression programs can evade tumor immune recognition. For instance, DUX4 expression blocks IFN-γ-mediated induction of MHC-I, demonstrating suppressed antigen presentation in DUX4-mediated IEV (Chew et al., 2019). Moreover, activation of β-catenin signaling promotes T cell exclusion from the melanoma microenvironment (Spranger et al., 2015), and LSD1 expression prevents antitumor immunity (Sheng et al., 2018). Several studies recently revealed that the signatures based on gene expression of tumor immune infiltration cells manifest potentially better prognostic values (Wu et al., 2019; Bao et al., 2020; Giri, 2020; Gao et al., 2021; Wang et al., 2022). However, studies to evaluate the value of immune evasion-related genes (IEVGs) in the prediction of the prognosis of patients with colon cancer are lacking.

In this study, we evaluated the expression of 182 IEVGs and identified 42 RFS-related IEVGs of patients with colon cancer from the training cohort. The immune evasion-related genes signature (IEVSig) was constructed by using 16 prognostic IEVGs screened from the Lasso Cox regression model and further validated in an external cohort. Functional enrichment and immune cell infiltration analyses were conducted to investigate the potential mechanism of IEV. Furthermore, genomic alteration and somatic mutation analyses were conducted to explore the relationship between genetic variation and IEV. At last, we assessed the prognostic value of IEVSig in response to cancer immunotherapy.

## Materials and Methods

### Data Collection

The entire sets of 182 IEVGs of cytotoxic T lymphocytes by using the genome-wide CRISPR screens across a panel of genetically diverse mouse cancer cell lines were obtained from a previous study (Lawson et al., 2020). Thereafter, we matched them to the human genes, which are listed in Supplemental Table S1. To observe IEV in colon cancer, by checking the database from the Gene Expression Omnibus (GEO) and The Cancer Genome Atlas (TCGA), the patients who met the following criterion were reserved: 1) have overall/recurrence-free survival (RFS) data and 2) have integral clinical data, such as AJCC TNM stage, age, sex, and MSI status. As a result, the gene expression profiling datasets of four cohorts, namely, GSE39582, GSE33113, GSE38832, and GSE39084, were downloaded from the GEO, whereas the TCGA-COAD cohort was obtained from TCGA public databases (Supplemental Table S2). The clinical data of these patients were collected, including TNM status, MSI status, stage, age, and gender (Supplemental Table S3). Tumor mutation burden (TMB), copy number variation (CNV) burden, loss of heterozygosity (LOH) score, and somatic mutation analysis were conducted between patients with low- and high-risk colon cancer of the TCGA-COAD cohort. Among them, GSE39582 was the training cohort, and others were validation cohorts.

### Construction and Validation of Immune Evasion-Related Genes Signature Based on the Prognostic Immune Evasion-Related Genes Signature

RFS rate was the primary endpoint in our study, so we screened the targeted genes based on the RFS-related IEVGs (|HR| > 1, *p* < 0.05) using univariate Cox regression for the 182 IEVGs in the GSE39582 training cohort. The RFS-related IEVGs were subjected to Lasso Cox regression analysis to establish the prognostic prediction model using the package of “glmnet” (version 4.1–3) (Friedman et al., 2010; Tu et al., 2020). The coefficients of the identified IEVGs in the Lasso Cox regression model were used to calculate the IEVSig, with the penalty parameter estimated via 10-fold cross-validation, and all the patients were divided into the high- and low-risk groups based on the median risk score. The formula was calculated as follows:

$$\mathrm{IEVSig}\;=\;\sum_{i=1}^{n}\beta i\;\;*expi$$

where βi is the coefficient of the identified IEVGs in the Lasso Cox regression model and *expi* is the normalized expression of the IEVGs.

### Immune Cell Infiltration Analysis and Gene Set Variation Analysis

To compare the differences in immune cell types between the high- and low-risk IEVSig groups, we used the CIBERSORT method, a deconvolution algorithm that uses support vector regression for calculating the detailed immune cell types in patients with colon cancer (Newman et al., 2015; Newman et al., 2019). For the current signatures with marker genes, we used the single simple gene set enrichment analysis (ssGSEA) method by the gene set variation analysis (GSVA) R package to calculate the enrichment score to represent the activity of these signatures in the patients with colon cancer.

### Functional and Pathway Enrichment Analyses

By using the “clusterProfiler” R package (version 4.2.2) (Yu et al., 2012), the Kyoto Encyclopedia of Genes and Genomes (KEGG) enrichment analysis was conducted in our study. Gene set enrichment analysis (GSEA) was used to explore the potential function and signaling pathway enrichment associated with the patients with high- and low-IEVSig colon cancer. Enrichment *p* values were based on 1,000 permutations and subsequently adjusted using the Benjamini–Hochberg (BH) method.

### Immune Evasion-Related Genes Signature in Cancer Immunotherapy

To further evaluate the potential value of IEVSig in cancer immune therapy, an immune-related cohort of advanced urothelial cancer with atezolizumab (n = 348) from the “IMvigor210” cohort (Mariathasan et al., 2018) as the immune-related validation cohort was utilized.

### Statistical Analysis

The Kaplan–Meier method was conducted to evaluate RFS differences between the high- and low-risk groups using “survminer” (version 0.4.9) and “survival” (version 3.2–13) packages. Then, we used the “surv-cutpoint” function of the “survminer” package to divide patients into two subgroups with the most significant statistical results. In addition, the receiver operator characteristic (ROC) curve was used to evaluate the accuracy of the prognostic prediction model using the R package “timeROC” (version 0.4) (Blanche et al., 2013). The Wilcox method was used to compare the differences in levels between two subgroups. IEVSig was validated in the validation cohorts. All the statistical analyses including univariate and multivariate Cox regression analyses, Lasso Cox regression analysis, Kaplan–Meier survival analysis, and ROC curve analysis were processed using R software (version 3.6.1). All reported *p* values were two-sided, and statistical significance was set at 0.05.

## Results

### Identification of Prognostic Immune Evasion-Related Genes Signature

**FIGURE 1:**
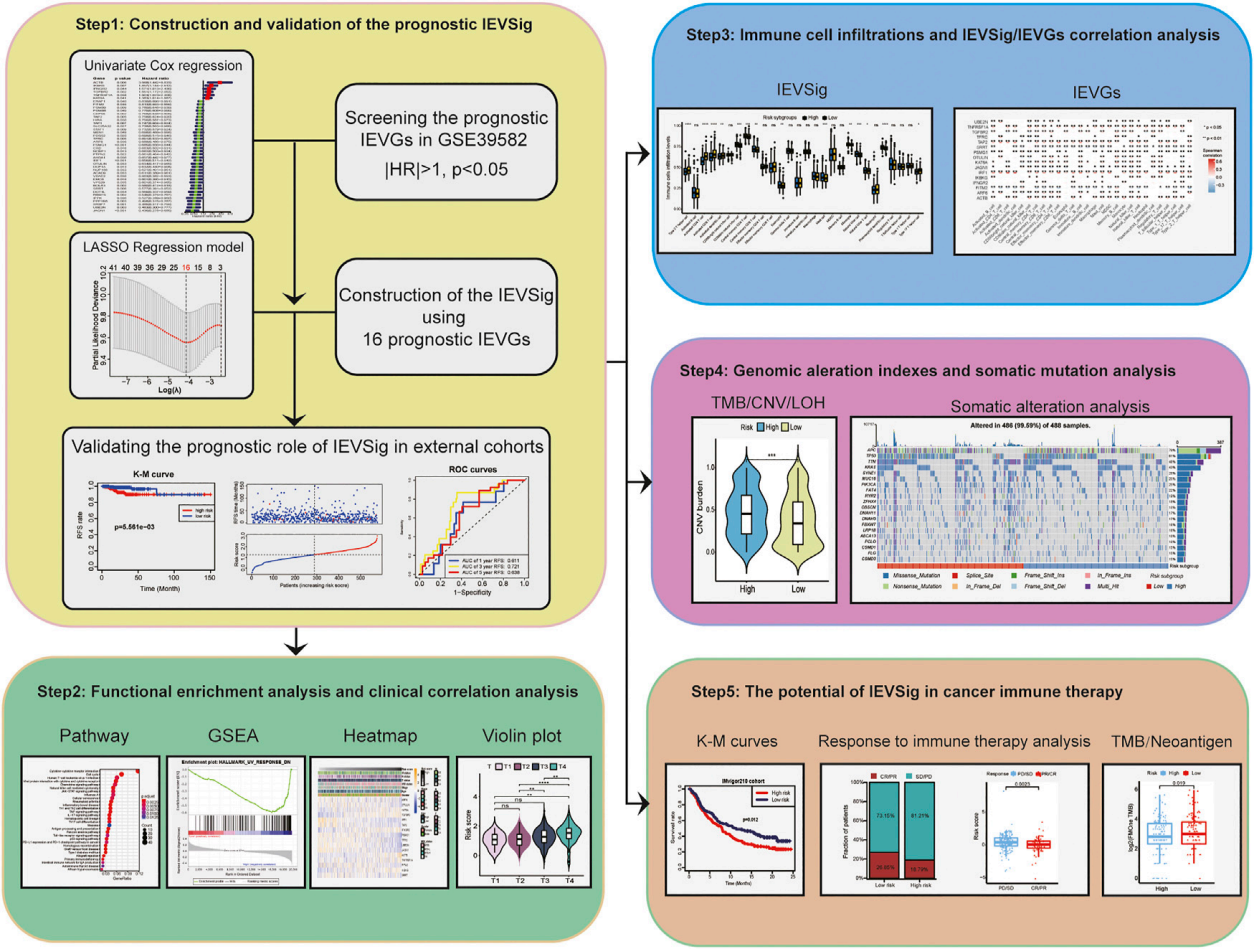
Workflow chart of our study.

**FIGURE 2:**
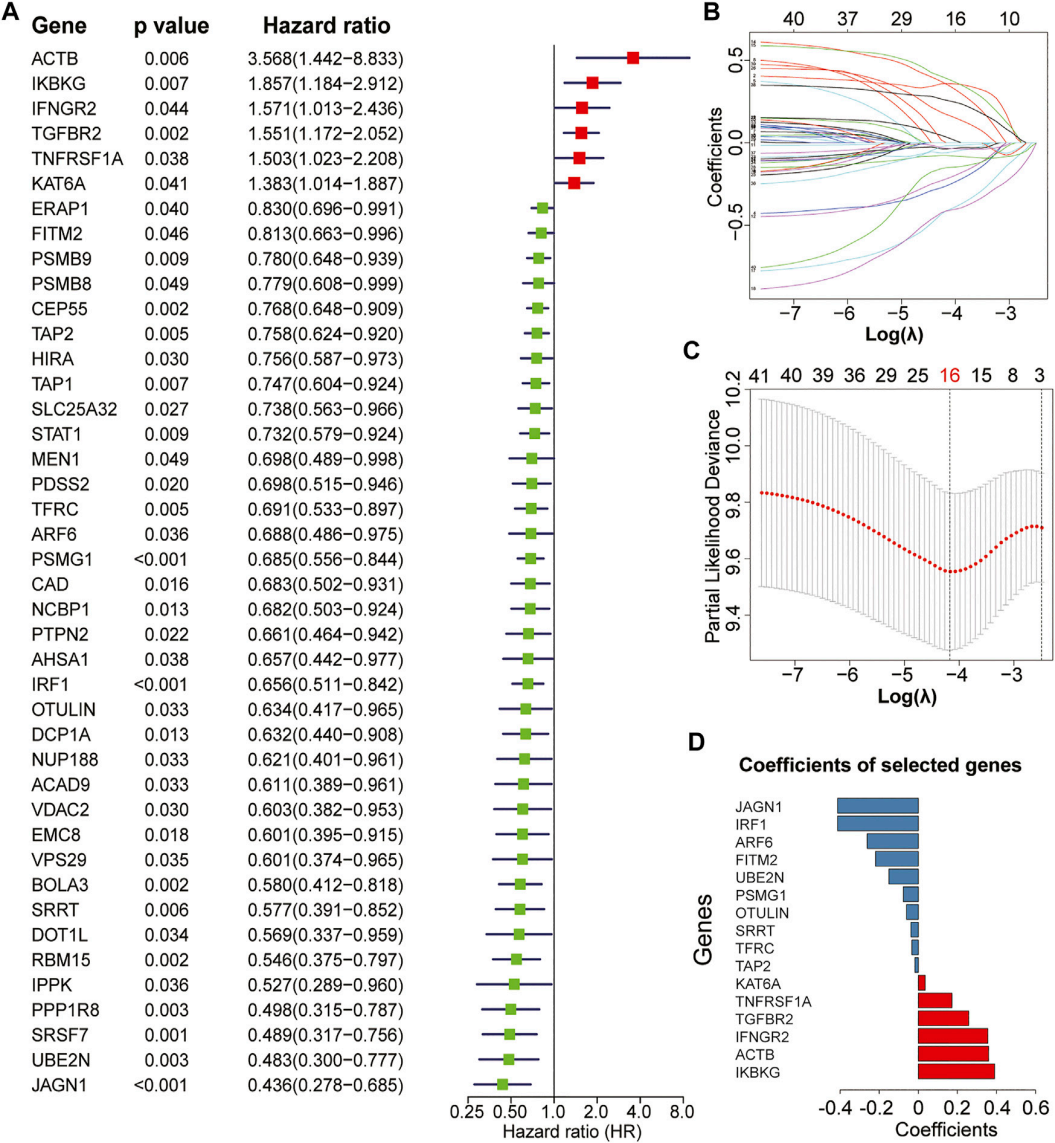
Establishment of the immune evasion-related genes (IEVG) signature model. **(A)** univariate Cox regression screening of the 42 recurrence-free survival-related IEVGs screened from 182 IEVGs in the GSE39582 training cohort. **(B)** The coefficient profiles of the 42 prognostic IEVGs in the Lasso regression model from the training cohort (GSE39582). **(C)** Partial likelihood deviance of IEVGs included in the Lasso regression model. **(D)** Coefficients of the 16 screened IEVGs in the Lasso Cox regression model.

The complete flowchart is shown in Figure 1. In our study, we used the GEO dataset GSE39582 gene expression profiling as a training cohort to identify prognostic IEVGs. Among 182 previously reported IEVGs in cancer, 42 IEVGs associated with RFS were identified through univariate Cox regression analysis in colon cancer (Figure 2A). Then, Lasso Cox regression analysis was used to establish the prognostic prediction model based on the 42 RFS-related IEVGs. The coefficients of the identified IEVGs are shown in Figure 2B. We calculated the partial likelihood deviance of IEVGs included in the Lasso regression model (Figure 2C). At last, 16 RFS-related IEVGs were identified, and the prognostic signature named IEVSig was further constructed. The detailed coefficients of the 16 screened IEVGs in the Lasso Cox regression model and the BH adjusted *p* value are shown in Figure 2D and Supplemental Table S4.

### Establishment and Validation of Immune Evasion-Related Genes Signature

**FIGURE 3:**
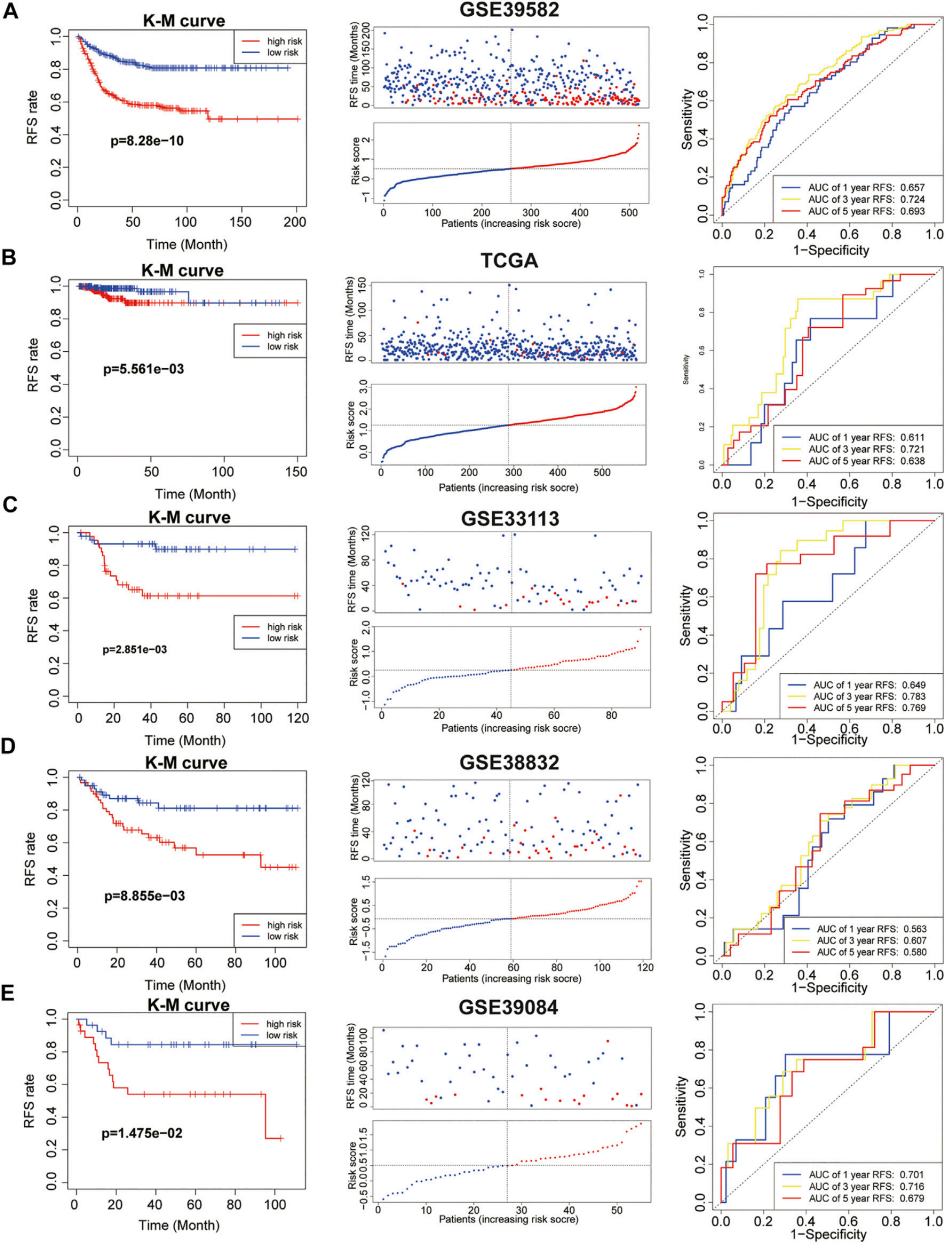
Immune evasion-related genes signature (IEVSig) was associated with CC survival. **(A–E)** Kaplan–Meier curves of recurrence-free survival according to IEVSig, CC patients’ distribution plots, and time-dependent receiver operator characteristic curves of IEVSig in the GSE39582 training cohort **(A)**, the Cancer Genome Atlas cohort **(B)**, GSE33113 **(C)**, GSE38832 **(D)**, and GSE39084 **(E)**.

We calculated IEVSig according to the coefficients of the 16 IEVGs identified in the Lasso Cox regression model and divided the patients into high- and low-risk groups based on the median risk score (Supplemental Table S5). In the GSE39582 training cohort, Kaplan–Meier curves and distribution plots showed that patients with colon cancer and high IEVSig had shorter RFS compared with patients with low IEVSig (*p* = 8.28e-10). In addition, time-dependent ROC curves were applied to estimate the signature, which indicated that the values of area under curves (AUCs) for 1-, 3-, and 5-year survival times were 0.657, 0.724, and 0.693, respectively (Figure 3A). In similar, Kaplan–Meier curves and distribution plots all showed that patients with colon cancer and high IEVSig had worse RFS in the TCGA, GSE33113, GSE38832, and GSE39084 validation cohorts (*p* < 0.05). In the TCGA cohort, ROC analysis showed that the AUCs for 1-, 3-, and 5-year survival times were 0.611, 0.721, and 0.638, respectively. In GSE33133, the AUCs for 1-, 3-, and 5-year survival times were 0.649, 0.783, and 0.769, respectively. In GSE38832, the AUCs for 1-, 3-, and 5-year survival times were 0.563, 0.607, and 0.580, respectively. In GSE39084, the AUCs for 1-, 3-, and 5-year survival times were 0.701, 0.716, and 0.679, respectively (Figures 3B–E). To detect the prior of IEVSig, the classical 20 colon prognosis-related signatures were collected from the MsigDB (http://www.gsea-msigdb.org/) and calculated via GSVA. Results showed that IEVSig had a higher ranking compared with other signatures via Cox regression analysis and time-ROC analysis (Supplemental Table S6).

In final, IEV Sig was significantly related to the prognosis of 17/30 cancers by using the pan-cancer cohorts (Supplemental Table S7).

### Comparison of Immune Evasion-Related Genes Signature With Clinicopathological Features of Patients With Colon Cancer

To further study the prognostic value of IEVSig and clinicopathological features, we conducted univariate and multivariate Cox regression analyses in the GSE39582 training cohort. Univariate Cox analysis revealed that the clinicopathological features, such as tumor stage and MMR status, and IEVSig could affect the prognosis of patients with colon cancer. Furthermore, multivariate Cox analysis indicated that IEVSig remained an independent prognostic factor (Table 1). In similar, IEVSig remained an independent prognostic factor in the TCGA_COAD validation cohort (Table 2).

**TABLE 1:** Univariate and multivariate Cox regression analysis in the GSE39582 cohort.

Variable	Univariate Cox Regression				Multivariate Cox Regression			
	HR	95% low	95% up	P	HR	95% low	95% up	P
Age (<60 vs. ≥ 60)	1.010	0.997	1.023	0.131	1.012	0.999	1.026	0.081
Gender (Female vs. Male)	1.288	0.924	1.796	0.136	1.313	0.932	1.850	0.120
Stage (I + II vs. III + IV)	2.275	1.629	3.177	0.000	2.060	1.454	2.919	0.000
MMR-status (pMMR vs. dMMR)	2.303	1.209	4.383	0.011	1.423	0.731	2.773	0.299
IEV Risk (Low vs. High)	2.936	2.050	4.206	0.000	2.771	1.883	4.077	0.000

**TABLE 2:** Univariate and multivariate Cox regression analysis in the Cancer Genome Atlas-COAD cohort.

Variable	Univariate Cox Regression				Multivariate Cox Regression			
	HR	95% low	95% up	P	HR	95% low	95% up	P
Gender (Female vs. Male)	0.779	0.330	1.841	0.569	0.965	0.369	2.521	0.941
Age (<60 vs. ≥ 60)	0.393	0.152	1.012	0.053	0.307	0.099	0.951	0.041
Stage (I + II vs. III + IV)	0.935	0.367	2.387	0.889	0.717	0.272	1.888	0.501
MSI-status (Non-MSI-H vs. MSI-H)	1.535	0.627	3.755	0.348	1.707	0.622	4.689	0.299
IEV Risk (Low vs. High)	3.758	1.375	10.268	0.010	4.412	1.385	14.055	0.012

**FIGURE 4:**
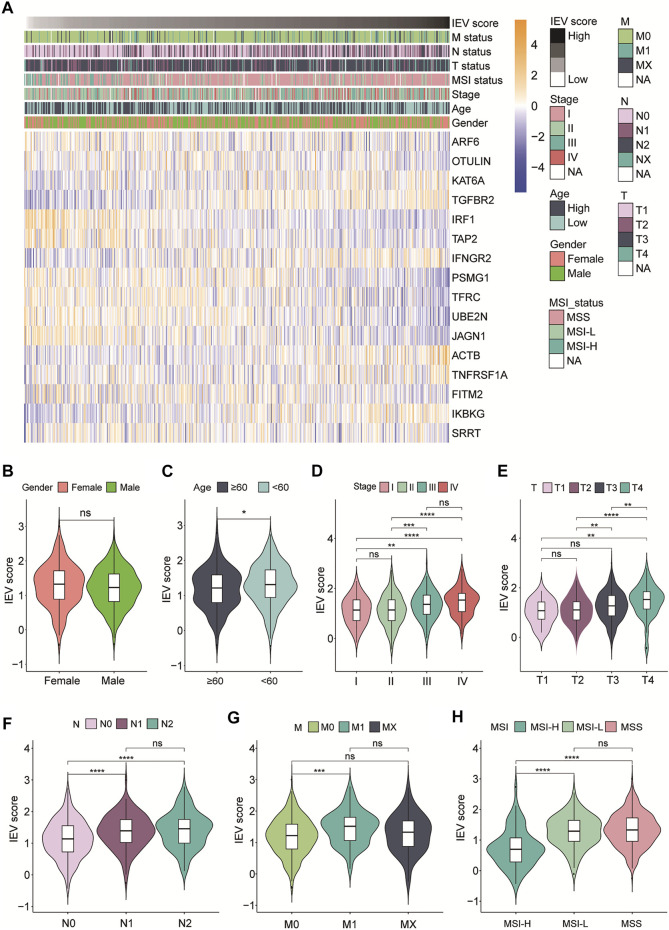
Immune evasion-related genes signature (IEVSig) was correlated with clinicopathological features of CC patients. **(A)** heatmap of the IEVSig consisting of 16 immune evasion-related genes ordered by the risk scores and its association with clinicopathological features including TNM status, MSI status, stage, age, and gender. **(B–H)** the violin plot representation of the correlations of the IEVSig and gender **(B)**, age **(C)**, stage **(D)**, T status **(E)**, N status **(F)**, M status **(G)**, and MSI status **(H)**.

Then, we evaluated the relevance between IEVSig and clinicopathological features of patients with colon cancer in the TCGA-COAD cohort. As shown in Figure 4A, the heatmap showed IEVSig ordered by the risk scores and the distributions of clinicopathological features, including TNM stage, MSI status, age, gender, and the expression levels of the 16 genes. In detail, the violin plot revealed that young patients had higher IEVSig than the old patients. In addition, the patients with high IEVSig had higher T status, higher N status, higher M status, and higher TNM stage than patients with low IEVSig (Figures 4B–H). Patients with high IEVSig had a more stable microsatellite status than those with low IEVSig (all *p* < 0.05).

### Functional Analysis of Immune Evasion-Related Genes Signature

**FIGURE 5:**
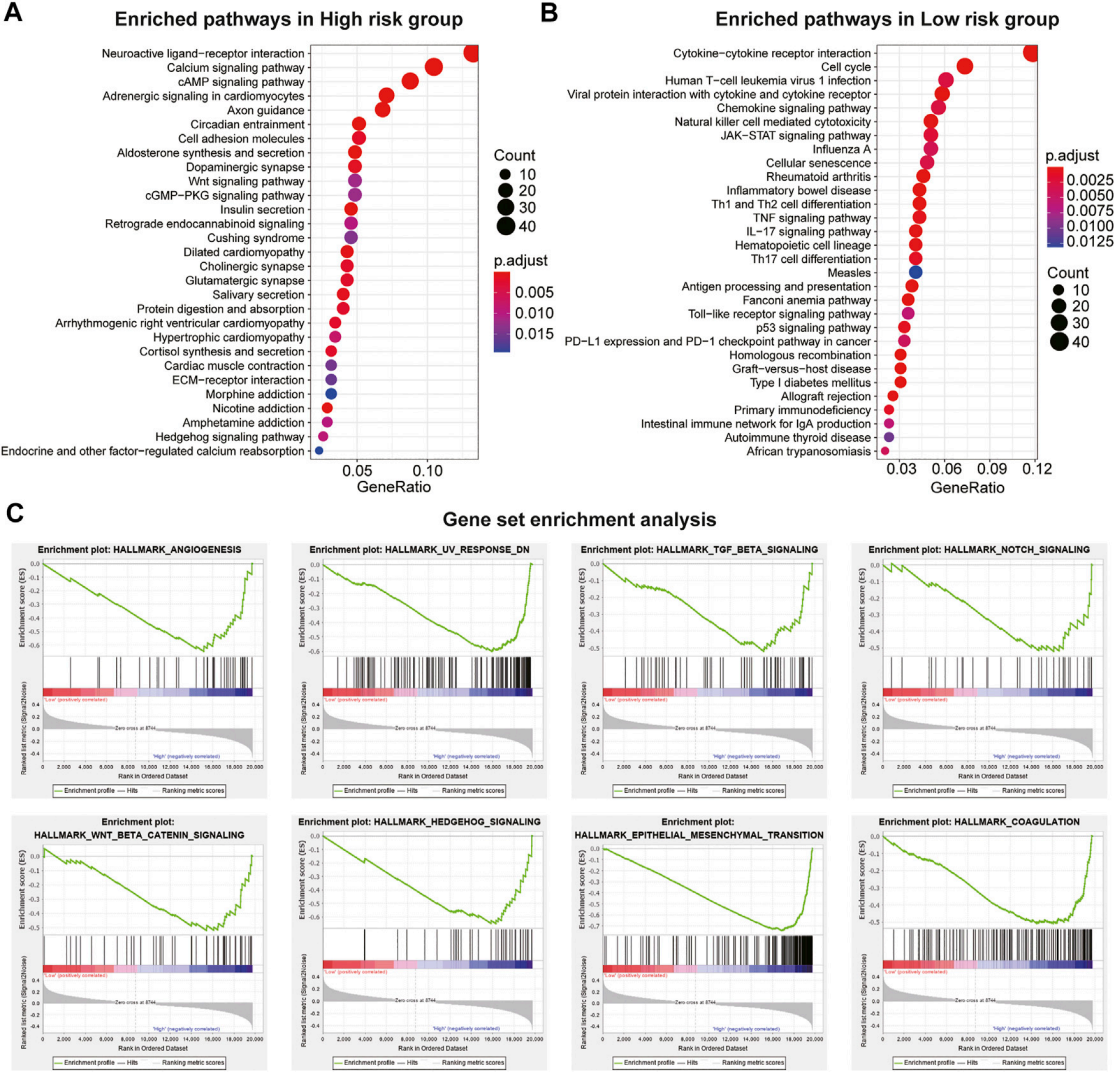
Pathway and hallmarks associated with the immune evasion-related genes signature. **(A)** top 30 enriched Kyoto Encyclopedia of Genes and Genomes (KEGG) pathways in high-risk CC subgroup vs. low-risk subgroup in the Cancer Genome Atlas (TCGA) cohort. **(B)** top 30 enriched KEGG pathways in low-risk CC subgroup vs. high-risk subgroup in the GSE39582 cohort. **(C)** significantly enriched hallmarks in high-risk subgroup CC patients using the GSEA method in the TCGA cohort.

We conducted KEGG and GSEA analyses to assess the potential function of the IEV-related gene signature in the training cohort (GSE39582). KEGG analysis showed the top 30 enriched KEGG pathways in patients with high-risk colon cancer compared with patients with low-risk cancer in the training cohort, which included neuroactive ligand–receptor interaction, calcium signaling pathway, cAMP signaling pathway, cell adhesion molecules, and the Wnt signaling pathway (Figure 5A and Supplemental Table S8). In addition, KEGG analysis showed that the enriched KEGG pathways in patients with low-risk colon cancer compared with the high-risk group were cytokine–cytokine receptor interaction, cell cycle, chemokine signaling pathway, NK cell-mediated cytotoxicity, and TNF signaling pathway (Figure 5B and Supplemental Table S8). GSEA analysis was conducted to analyze the pathways enriched in the patients with high-risk colon cancer. “angiogenesis,” “response_dn,” “tgf_beta_signaling,” “notch_signaling,” “coagulation,” “wnt_beta_catenin_signaling,” “hedgehog_signaling,” AND “epithelial_mesenchymal_transition” were obviously enriched in the patients with high-risk colon cancer (Figure 5C).

### Immune Cell Infiltration Levels Between Distinct Immune Evasion-Related Genes Signature Groups

**FIGURE 6:**
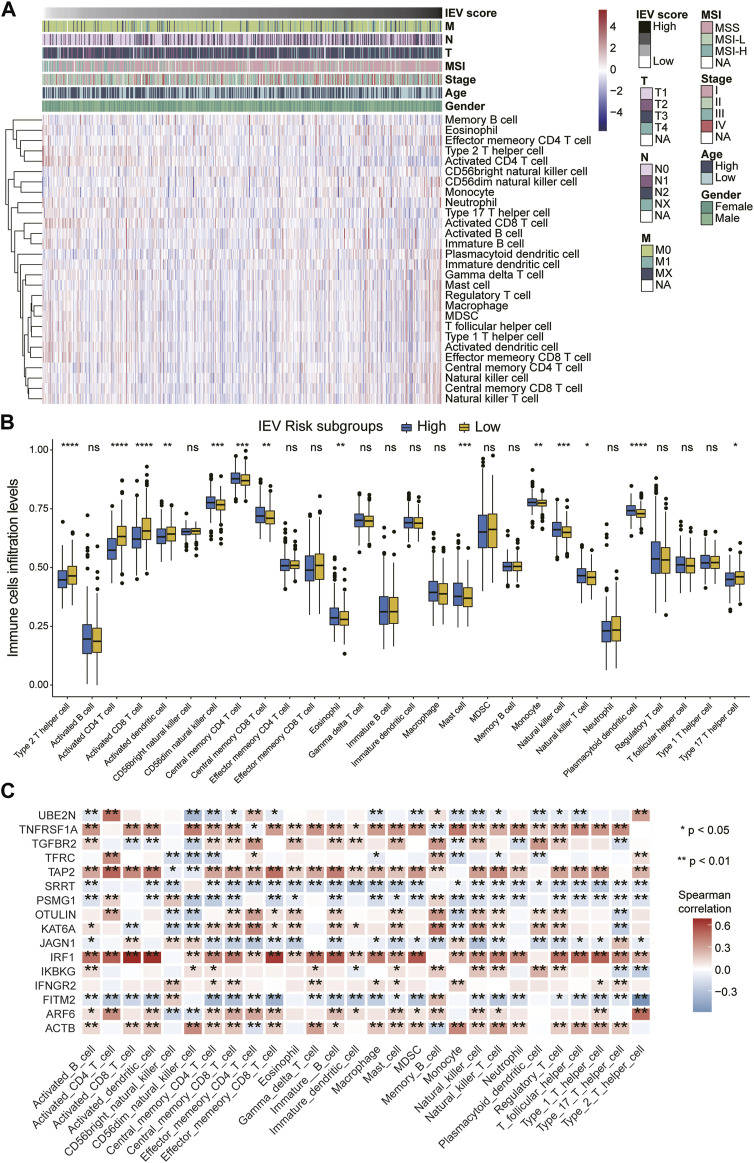
orrelations between Immune evasion-related genes signature and immune cell infiltration levels of CC patients. **(A)** heatmap of the 28 immune cell infiltration levels ordered by the risk scores and their association with clinicopathological features including TNM status, MSI status, stage, age, and gender in the Cancer Genome Atlas (TCGA) cohort. **(B)** boxplot represents the 28 immune cell infiltration levels between low- and high-risk CC subgroups in the TCGA cohort. **(C)** the heatmap shows the Spearman correlations between the 28 immune cell infiltration levels and 16 screened immune evasion-related genes in the TCGA cohort.

We estimated the relative proportion of the 28 immune cells for each patient with colon cancer with high or low IEVSig using CIBERSORT in the TCGA-COAD cohort. The infiltration levels of 28 immune cells of patients with different risk scores are shown in Figure 6A. CD56dim NK cell, central memory CD4 T cell, central memory CD8 T cell, eosinophil, mast cell, monocyte, NK cell, NK T cell, and plasmacytoid dendritic cell obviously increased in the high-risk groups than in the low-risk groups. However, the expression levels of type 2 T helper cells, activated CD4 T cells, activated CD8 T cells, activated dendritic cells, and type 17 T helper cells obviously decreased in the high-risk groups (Figure 6B). The heatmap showed that the 16 IEVGs of IEVSig were significantly correlated with the 28 immune cell infiltration levels (Figure 6C).

### Genomic Alteration and Somatic Mutation of Immune Evasion-Related Genes Signature Groups

**FIGURE 7:**
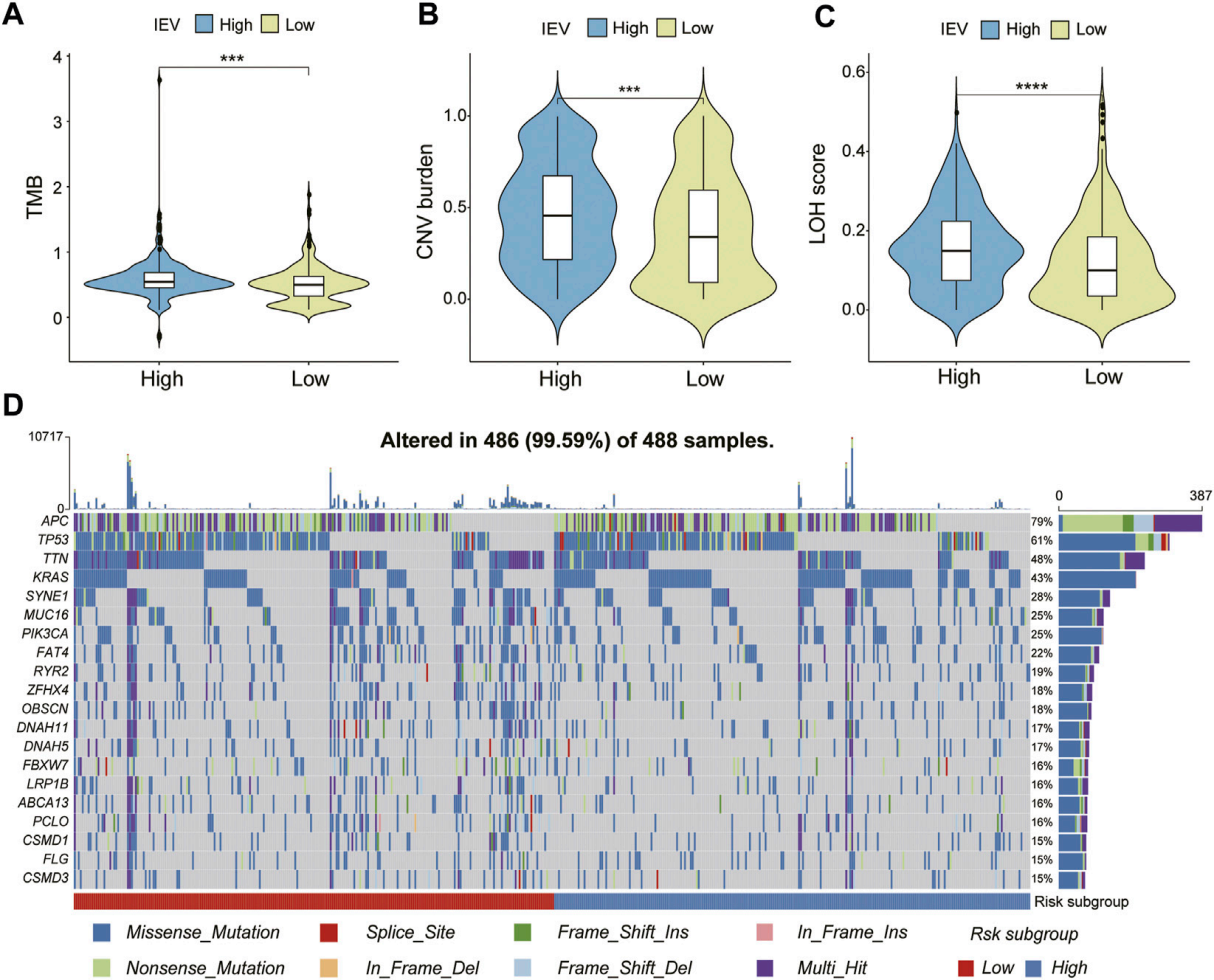
Genomic alteration and somatic mutation analysis in the Cancer Genome Atlas cohort. **(A–C)** tumor mutation burden, copy number variation burden, and loss of heterozygosity score were compared between low- and high-risk subgroups. **(D)** somatic mutation landscape of top 20 frequent SMGs between low- and high-risk CC subgroups.

We evaluated the genomic alteration and somatic mutation status between patients with high and low IVESig in the TCGA-COAD cohort. As shown in Figure 7A, patients with high IVESig had higher TMB, CNV burden, and LOH score than those with low IVESig. Moreover, the various types of common gene mutations of the two IEVSig groups are presented in Figure 7B.

### Prediction of Immune Evasion-Related Genes Signature in Guiding Cancer Immunotherapy

**FIGURE 8:**
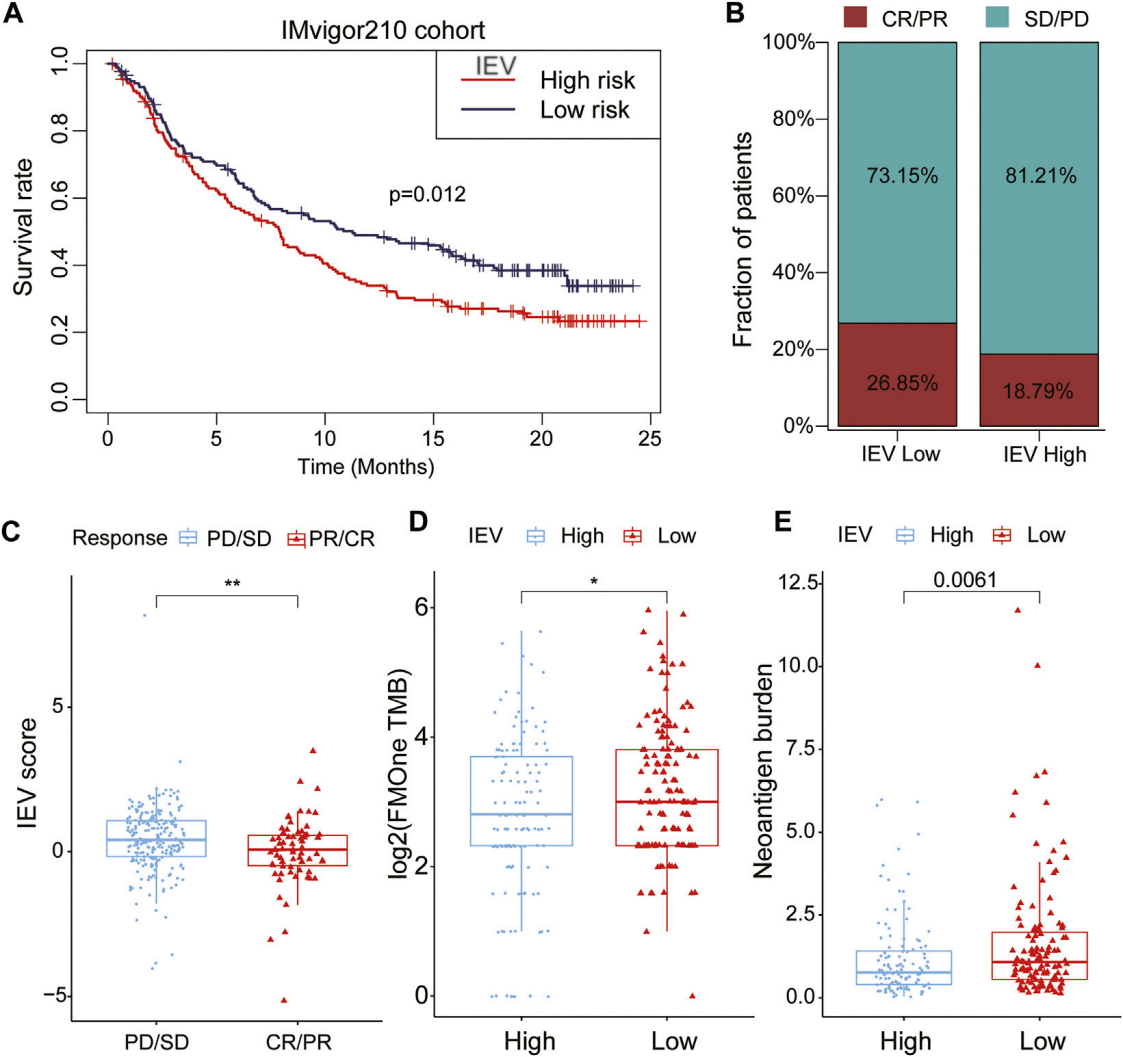
Potential of Immune evasion-related genes signature (IEVSig) in guiding cancer immune therapy. **(A)** Kaplan–Meier curves of the low- versus high-risk subgroup in the IMvigor210 cohort (anti-PD-L1 cohort). **(B)** the proportion of immune therapy response to anti-PD-L1 treatment in low- versus high-risk subgroups. CR, complete response; PR, partial response; SD, stable disease; PD, progressive disease. **(C)** distribution of IEVSig score between distinct immune therapy response statuses (PD/SD vs. CR/PR). **(D,E)** tumor mutation load **(D)** and neoantigen burden **(E)** in the anti-PD-L1 cohort were compared between distinct IEVSig subgroups.

To assess the potential values of IEVSig in guiding cancer immunotherapy, we obtained the Kaplan–Meier curves and immunotherapy response by using the IMvigor210 immunotherapeutic cohort. The Kaplan–Meier curves showed that patients with high IEVSig had shorter survival (Figure 8A, *p* = 0.012) than those with low IEVSig. In addition, patients with high IEVSig had a higher proportion of progressive disease or stable disease after receiving immunotherapy than patients with low IEVSig (Figures 8B, C). Furthermore, patients with low IEVSig had higher tumor mutation load and neoantigen burden, which indicated an improved immunotherapy response (Figures 8D, E).

## Discussion

Colon cancer has high mortality rates across the world, despite significant advances in diagnosis and therapy (Bray et al., 2018; Dekker et al., 2019). Besides genetic aberrations, posttranscriptional alterations are also involved in the regulation of colon cancer development (García-Cárdenas et al., 2019; Gao et al., 2021). Given the heterogeneity of cancer, patients with colon cancer manifest distinct prognoses to various therapeutic approaches. IEV of TME plays a key role in the development and progression of colon cancer, so analysis of IEV-related genes will help us fully understand the pathogenicity and accurately predict the prognosis of individual patients.

Increasing evidence has shown that the TME is intricately related to cancer development and progression, guiding clinical therapy (Fang and Declerck, 2013; Binnewies et al., 2018). Cancer cells acquire phenotypic changes to evade recognition and destruction by effector cells of the immune system to complete IEV. Therefore, an IEV-related gene signature must be constructed to predict the prognosis of patients with colon cancer.

In this study, we constructed a novel prognostic model, named IEVSig, which consisted of 16 IEV-related genes. This model divided the patients into high- and low-risk groups according to the survival outcome. Patients in the high-IEVSig group exhibited poor prognosis, whereas patients in the low-IEVSig group showed prolonged survival time. In total, 16 IEV-related genes were identified in our study. TGFBR2 (transforming growth factor, beta receptor II) is a protein-coding gene that binds to TGF-β. TGF-β is a potent immunosuppressor that is associated with tumor escape from the surveillance of the host immune, and it promotes tumor progression. Therefore, blockade or insensitivity of TGF-β would be a potential therapeutic strategy to enhance antitumor immunotherapy (Zhang et al., 2006; Wang et al., 2010; Tauriello et al., 2018). Rachel A Burga et al. (Burga et al., 2019) reported a new strategy to engineer TGFβ receptors of NK cells, which enable inhibitory TGFβ signals to convert to activating signals and overcome TGFβ-mediated IEV. A previous study demonstrated that upregulation of IRF1 inhibits the progression of CRC by regulating interferon-induced proteins (Xu et al., 2021). Wang et al. (Wang et al., 2020) also found that Mettl3 or Mettl14 loss promotes IFN-γ-Stat1-Irf1 signaling by stabilizing Stat1 and Irf1 mRNA via Ythdf2 to enhance the response to anti-PD-1 treatment. Another study revealed that increased Arf6 activity can enhance cell migration and invasion *in vitro* and increase metastasis of transplanted tumor cells in mice (Lüttgenau et al., 2021). The enhancement of the ARF6-based pathway and its activation by external ligands may promote tumor cell motility, PD-L1 dynamics, and IEV of pancreatic cancer (Hashimoto et al., 2019). Another study found that long noncoding RNA (lncRNA) DANCR binds with KAT6A to affect the acetyltransferase activity of KAT6A, thereby influencing the expression of KAT6A target genes to promote the development and progression of colon cancer (Lian et al., 2020). All the abovementioned IEV-related genes demonstrated that the IEVGs identified in our study were linked to the pathogenesis and escape of the host immune system in colon cancer.

Immune checkpoint blockade (ICB) treatment has emerged as the new therapeutic strategy for metastatic tumors. Previous research revealed that the high microsatellite instability (MSI-H) status and elevated mutational load can elevate sensitivity to ICB treatment (Le et al., 2017). Therefore, we conducted immune cell infiltration and genetic variation analyses to further evaluate the characteristics of TME and the response to immunotherapy. Immune cell infiltration analysis showed the significantly different infiltrated immune cells between the high- and low-IEVSig groups. Nine immune cells increased in the high-IEVSig group, whereas five immune cells increased in the low-IEVSig group. Genetic variation analysis showed that patients with low IEVSig had higher tumor mutation load and neoantigen burden and better response to immunotherapy than patients with high IEVSig, which indicated the correlation between IEV-related genes and TME.

Nevertheless, some limitations should be considered in our study. First, the study was conducted based on the public datasets, so several potential biases may exist. Second, the biological functions and molecular mechanisms of 16 IEV-related genes in CRC need to be further evaluated. At last, the identified 16 IEV-related genes require experimental verification and validation in more cohorts.

## Conclusion

In conclusion, we constructed an IEV-related signature IEVSig to predict prognosis in patients with colon cancer. Results showed that the high-IEVSig group had a significantly poor RFS than the low-IEVSig group. We found that the immune cell infiltration levels, tumor mutation load, neoantigen burden, and responses to immune therapy treatment were significantly different between groups, which indicated the interaction of IEV-related genes with TME. The IEV-related gene signature is an effective prediction marker to offer insights into therapeutic strategies.

## Data Availability

The original contributions presented in the study are included in the article/Supplementary Material, and further inquiries can be directed to the corresponding author.
